# 436. SARS-CoV-2 Seropositivity and Association with Dengue Severity in Adults

**DOI:** 10.1093/ofid/ofad500.506

**Published:** 2023-11-27

**Authors:** Megha Priyadarshi, Manish Soneja, Saurav Sekhar Paul, Ayush Agarwal, Praveen Kumar Tirlangi, Shilpa Sachan, Nimesh Gupta, Naveet Wig

**Affiliations:** All india institute of medical sciences, Delhi, Delhi, India; All India Institute Of Medical Sciences, Delhi, Delhi, India; All india Institute of Medical Sciences, New Delhi, delhi, Delhi, India; All India Institute of Medical Sciences, Delhi, Delhi, India; Kasturba medical college, Manipal, Karnataka, India; National Institute of Immunology, Delhi, Delhi, India; National Institute of Immunology, Delhi, Delhi, India; All India Institute of Medical Sciences, Delhi, Delhi, India

## Abstract

**Background:**

The effect of SARS-CoV-2 on the pathogenesis and virulence of the dengue virus is unknown. The cross-reactivity of the immune responses in these infections is an emerging concern, as it may influence the clinical outcomes by establishing cross-immunity or antibody-dependent enhancement. This study evaluated the association between SARS-CoV-2 seropositivity and clinical severity in dengue patients.

Fig 1

**Figure:**
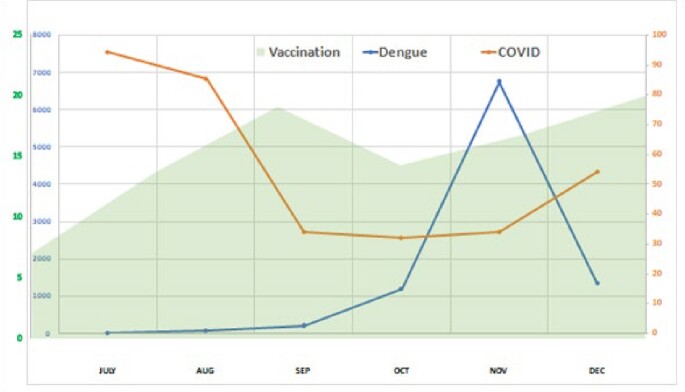

SARS-CoV-2 cases in the region where the study was conducted and adults with dengue presenting to the study site

**Methods:**

We performed a cross-sectional study on confirmed dengue cases admitted at a tertiary care hospital in North India. Baseline demographic data, clinical severity, past history of symptomatic COVID infection, and COVID-19 vaccination were noted. IgG antibodies against the receptor binding domain (RBD) of the SARS-CoV-2 virus were measured in all patients. The association between the SARS-CoV-2 antibody titer and severity of dengue was statistically analyzed using one-way ANOVA - Kruskal Wallis followed by Dunn's multiple comparisons, with a p-value less than 0.05 considered statistically significant.

**Results:**

A total of 62 confirmed dengue patients were enrolled in the study (September 2021–December 2021). The median age of the study population was 27 years (IQR: 21–35 years), with a male preponderance of 64.5%. According to WHO dengue severity criteria, 30.6% of patients had dengue without warning signs, 50% had dengue with warning signs, and 19.3% had features suggestive of severe dengue. All patients were seropositive for IgG COVID antibodies, with a median titer of 2700 AU/ml (IQR 900–8100). We observed no statistically significant difference in overall SARS-CoV-2 antibody responses among the three dengue severity groups (p-value 0.9).

Table 1

**Figure:**
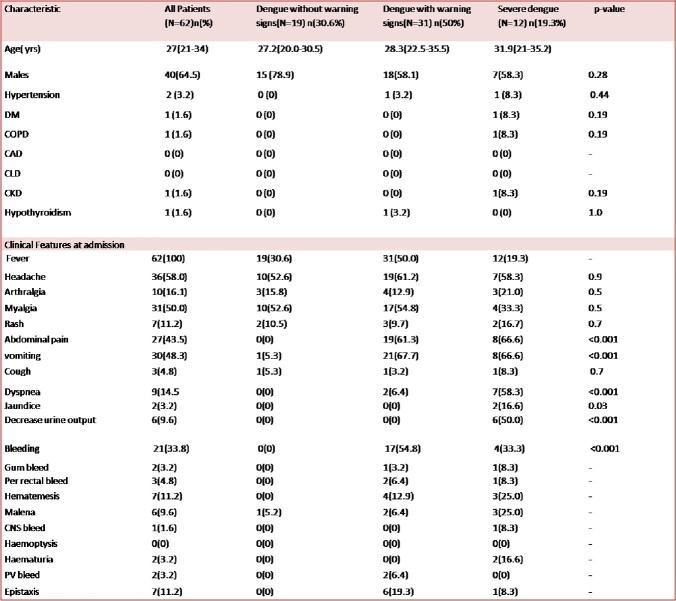

Demographic and clinical characteristics of study population and comparison of presentation with varying severity of dengue

Table 2

**Figure:**
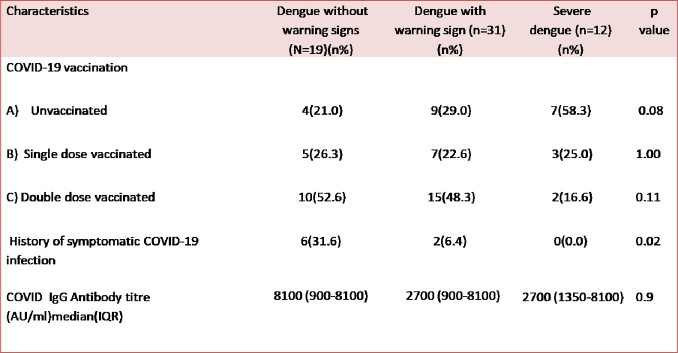

Association of SARS CoV2 infection and COVID vaccination with dengue severity

Fig 2

**Figure:**
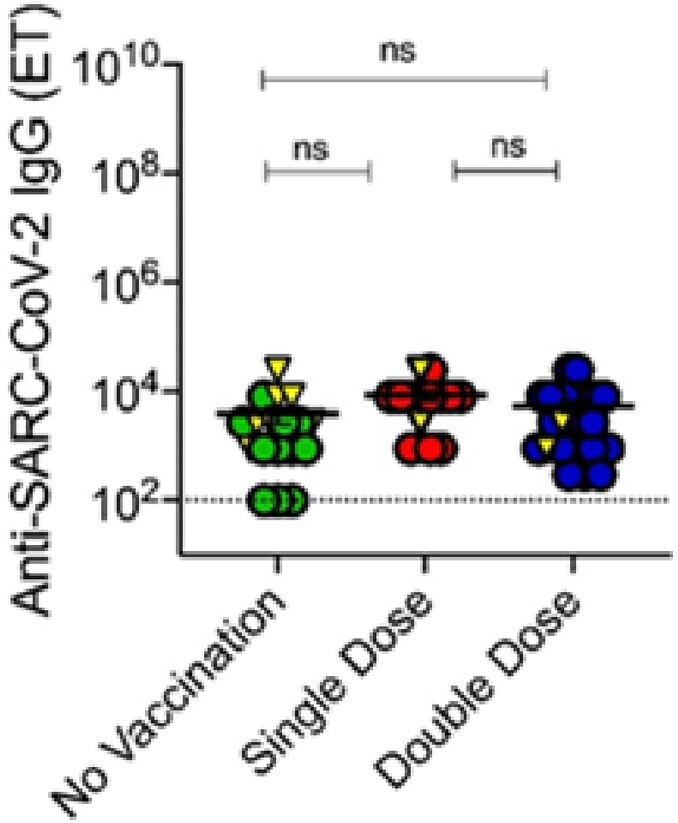

Effect of COVID-19 vaccination on SARC-CoV-2 specific antibodies in acute dengue patients. Acute dengue patients categorized as non-vaccinated (Green circles, N=12), single (Red circles, N=12) and double dose vaccinated (Blue circles, N=24) with SARS-CoV-2 vaccines. Yellow triangle represents severe dengue (N=11). (A-B) Graph demonstrates the spread of SARS-CoV-2 binding IgG in three groups respectively. Dotted line represents the cut-off for IgG positivity

**Conclusion:**

This study concludes that SARS-CoV-2 seropositivity is not associated with clinical severity in dengue patients. This may imply that past infection with COVID or vaccination against COVID does not alter the severity of dengue.

Fig 3

**Figure:**
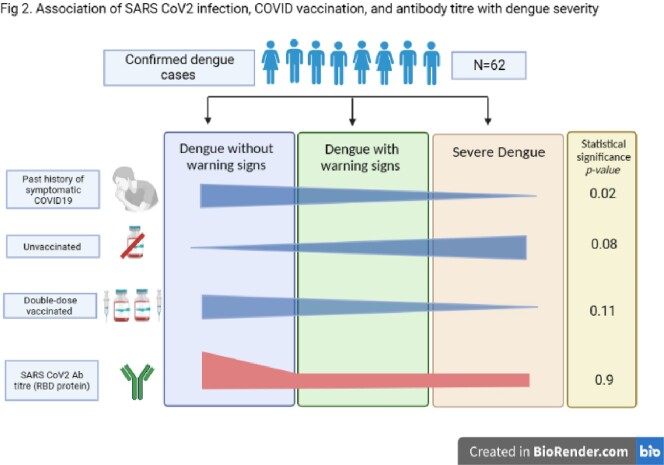

Association of SARS CoV2 infection COVID vaccination and COVID antibody titer with dengue severity

**Disclosures:**

**All Authors**: No reported disclosures

